# Semi-synthetic reference diets containing crystalline amino acids at 120% of recommendations for adult dogs are not appropriate for estimating the protein quality of ingredients used in extruded diets

**DOI:** 10.1093/jas/skaf306

**Published:** 2025-08-30

**Authors:** Michelina Crosbie, James R Templeman, Julia G Pezzali, Alexandra Rankovic, Glenda Courtney-Martin, Crystal Levesque, Leslie Hancock-Monroe, Preston R Buff, Daniel A Columbus, Adronie Verbrugghe, Anna K Shoveller

**Affiliations:** Department of Animal Biosciences, University of Guelph, Guelph, ON, N1G 2W1, Canada; Department of Animal Biosciences, University of Guelph, Guelph, ON, N1G 2W1, Canada; Department of Animal Biosciences, University of Guelph, Guelph, ON, N1G 2W1, Canada; Department of Animal Biosciences, University of Guelph, Guelph, ON, N1G 2W1, Canada; Department of Nutritional Sciences, University of Toronto, M5S 1A8Canada; Department of Animal Sciences, South Dakota State University, 57007United States; The J.M. Smucker Co., Orrville, OH, 44667-0280United States; The J.M. Smucker Co., Orrville, OH, 44667-0280United States; Department of Animal and Poultry Science, University of Saskatchewan, Saskatoon, SK, S7N 5A8Canada; Prairie Swine Centre Inc., Saskatoon, SK, S7K 3J4, Canada; Department of Clinical Studies, Ontario Veterinary College, University of Guelph, Guelph, ON, N1G 2W1Canada; Department of Animal Biosciences, University of Guelph, Guelph, ON, N1G 2W1, Canada

**Keywords:** chicken meal, canine nutrition, indicator amino acid oxidation, methionine, metabolic availability, peas

## Abstract

Metabolic availability (**MA**) of amino acids (AA) in feedstuffs has been assessed in pigs and humans using the indicator amino acid oxidation (**IAAO**) method. Our lab previously reported higher methionine (**Met**) content but lower MA in chicken meal (**ChM**) as compared to peas. However, the MA of met in peas or ChM was not quantified due to the absence of a crystalline AA reference diet, assumed to have 100% MA. This study aimed to quantify the MA of Met in peas and ChM compared to a semi-synthetic crystalline AA reference diet. A partially replicated 7 × 7 Latin square design was conducted in nine mixed-breed dogs [2.5 yrs old; 26.7 ± 2.7 kg body weight (**BW**)] fed to maintain ideal BW. Dogs received all seven dietary treatments: corn starch and barley-based reference diet supplying AA primarily as crystalline AA with Met at 19%, 40%, or 63% of requirement (BAS19, BAS40, BAS63, respectively), pea- (PEA63) and ChM- (ChM63) based diets with Met at 63%, and blends of BAS19 with peas (PEA40) or ChM (ChM40), both with Met at 40% of requirement. This created three graded levels of Met for ChM, peas, and BAS reference diets, permitting a slope ratio approach to quantify MA with the BAS diet as the common first point. Diets were isoenergetic, isonitrogenous, and met ≥ 120% of Association of American Feed Control Officials (**AAFCO**) AA recommendations for adult dogs (excluding Met). After 2 (**D2**) and 6 (**D6**) d of diet adaptation, IAAO was performed using L-[1-^13^C]-phenylalanine (**Phe**, 99%). Enrichment of ^13^CO_2_ in collected breath samples was measured using isotope-ratio mass spectrometry to calculate oxidation (F^13^CO_2_/kg BW/h). Data were analyzed using proc GLIMMIX with dog and period as random effects, and diet, % Met provided, IAAO day, and their interactions as fixed effects. There was no effect of day on MA, indicating the standard 2-d diet adaptation appears sufficient for determining the MA of AA using IAAO. Overall, ChM had the lowest oxidation level, and the MA of peas was 66% and 51% of ChM on D2 and D6, respectively. Additionally, the BAS diet had no response, suggesting another limiting AA in the BAS diet.

## Teaser

Using the indicator amino acid oxidation technique, methionine metabolic availability was lower in peas than in chicken meal in adult dogs. However, the lack of response to the semi-synthetic crystalline amino acid reference diet providing ≥ 120% of published amino acid recommendations (except methionine) for adult dogs suggests another limiting amino acid, beyond methionine.

## Introduction

The indicator amino acid oxidation (**IAAO**) technique is a minimally invasive method that has been used to determine indispensable amino acid (**AA**) requirements in humans, pigs, dogs, and cats (Kim et al., 1983; Shoveller et al., 2017; Mansilla et al., 2018, 2020; Szwiega et al., 2023; Pezzali et al., 2024). Additionally, in humans and pigs, IAAO methodology has been used to determine the metabolic availability (**MA**) of AA in ingredients (Moehn et al., 2005; Fakiha et al., 2020; Tansil et al., 2022). MA studies, as opposed to requirement studies, seek to understand the portion of AA provided by an ingredient that is not only digestible but is also available for protein synthesis (Moehn et al., 2005). To determine the MA of AA in ingredients using the IAAO technique, experimental diets must be designed to contain at least three levels of the AA of interest provided below the animal’s requirement, with all other indispensable and related AA [e.g., cysteine (**Cys**) in the case of methionine (**Met**)] being provided in excess of the animal’s requirement (Moehn et al., 2005). This allows for a slope ratio approach to be used to calculate the MA of the test AA in the test ingredient compared to a reference protein considered to have 100% MA of AA (Moehn et al., 2005). Previous work determining MA of AA in swine typically used a reference diet providing all AA as free crystalline AA in a mash-type diet (Moehn et al., 2005; Tansil et al., 2022). However, over 60% of dogs consume extruded kibble as their primary diet and extrusion can both decrease the digestibility of AA in animal proteins and increase it in plant proteins (Dodd et al., 2020; Cargo-Froom et al., 2023b; Geary et al., 2023). We previously sought to determine the MA of Met in both peas and chicken meal (**ChM**) using ChM as a reference protein that could be extruded into a reference diet. However, the MA of Met in ChM was less than that of peas, likely due to heat damage to the Met content in ChM during both rendering and extrusion (Crosbie et al., 2024). The application of heat and pressure to process pet foods during extrusion may improve or reduce the digestibility of protein and AA, depending on how processing parameters are set (Awonorin et al., 1995; Tran et al., 2008). Additionally, it has been widely established that, in general, animal proteins have a greater variability in AA content and digestibility compared to plant proteins (Johnson et al., 1998; Bednar et al, 2000; Reilly et al., 2020). Therefore, exploring how to determine the MA of AA in ingredients in extruded diets while ensuring the base of the reference diet undergoes similar processing and the protein content of the reference diet undergoes minimal heat treatment is warranted.

Both peas and ChM are commonly used plant and animal protein sources in extruded dog foods (Faber et al., 2010; Plantz, 2017; Mansilla et al., 2019). However, while both ingredients are high in crude protein, they vary drastically in total dietary fiber content, with ChM containing negligible amounts and peas containing approximately 24% to 36% total dietary fiber on a dry matter basis including oligosaccharides (National Research Council, 2006; Reilly et al., 2020). Typically, ileal AA digestibility studies use a dietary adaptation period of 5 to 8 d prior to sampling to ensure animals are adapted to differing total dietary fiber and other macronutrient contents of the diet that impact AA digestibility (Bednar et al., 2000; Crosbie et al., 2020; Cargo-Froom et al., 2023a). Previous work in humans and pigs has shown that 2-d of dietary adaptation is sufficient for determining both AA requirements and a repeatable oxidation response in IAAO studies (Moehn et al., 2004; Elango et al., 2009; Szwiega et al., 2023). However, these studies used crystalline AA (Moehn et al., 2005; Shoveller et al., 2017; Tansil et al., 2022; Pezzali et al., 2024), and it is unknown whether the length of adaptation to different total dietary fiber contents would alter the MA of protein-bound AA in ingredients.

Therefore, the objectives of this study were to determine the MA of Met in both peas and ChM using a semi-synthetic crystalline AA reference diet and to determine if the MA of Met in these ingredients would differ when IAAO was conducted after the standard 2-d diet adaptation versus a 6-d diet adaptation. We hypothesized that 1) the MA of Met in peas would be greater than ChM based on previous work (Crosbie et al. 2024); and 2) the MA of Met would be reduced after a longer adaptation due to the higher dietary fiber content in peas compared to ChM.

## Materials and Methods

The experimental protocol and study design were reviewed and approved by the University of Guelph Animal Care Committee (AUP#4531). Handling and care of the animals were in accordance with the Canadian Council on Animal Care Guidelines (CCAC, 2020).

### Animals, housing, and acclimation to oxidation chambers

Nine neutered male mixed-breed hound dogs [2.5 yrs old; 26.7 kg ± 2.7 kg body weight (**BW**)] from Marshall BioResources (Waverly, NY, United States of America) were used in this study. All dogs resided at the University of Guelph Central Animal Facility (Guelph, ON, Canada). Seven dogs were single-housed, and one pair of dogs was housed together in kennels (3.7 m length, 2.0 m width, and 2.0 m tall) with visual and nose-to-nose contact. Constant environmental conditions were maintained in the kennel room with a mean temperature of 20.8 °C, a mean relative humidity of 59.0 % and a 12 h:12 h light:dark lighting schedule. Dogs had unlimited access to rubber and nylon toys and water in their kennels, and all dogs received 20 min of supervised outdoor walks 6 d/wk, unless the weather was poor, in which case dogs were walked indoors. All dogs had previously been adapted to eating multiple meals in crates, according to Crosbie et al. (2024) and Shoveller et al. (2017).

### Diets and study design

Three extruded kibble diets were formulated to determine the MA of Met in peas and ChM (Table 1). These included a corn starch and barley-based diet (**BAS**) providing Met at 19% of its requirement for Labrador Retrievers (**BAS19**: 0.10% dry matter (**DM**), Table 2; Mansilla et al., 2020), a ChM and lamb-based diet (**ChM63**), and a chipped green pea and lamb-based diet (**PEA63**) both providing Met at 63% of its requirement (0.32% and 0.33% DM, respectively; Mansilla et al., 2020). All protein-containing ingredients were added at the expense of corn starch to achieve pre-determined targets for Met content, but not crude protein content (Table 2). All diets were formulated to be isoenergetic (3,500 kcal/kg as-fed). However, after the formulation and production of the test diets were complete, the metabolizable energy (ME) content was calculated using the modified Atwater equation. These calculations revealed that the ME content of the PEA63 and ChM63 diets was 4.7% and 4.5% higher, respectively, compared to the ME content of the BAS19 diet. We did not consider this a significant difference among diets. Apart from fiber, all other nutrients were formulated to be held constant across all three diets. Fiber was not held constant due to the naturally high fiber content of peas, as fiber is an inherent difference between animal-based and pulse-based ingredients (Silvio et al., 2000). Lamb meal and deboned lamb were provided in both the ChM63 and PEA63 diets at the same inclusion levels (15.0% and 9.5% as-fed, respectively). Barley was included at similar levels across all diets, with the BAS19 diet containing 25.85% barley (compared to 25.0% in the ChM63 and PEA63 diets) to improve the extrusion of a primarily corn-starch-based BAS diet, thereby making it semi-synthetic. These ingredients were used due to their inherently low Met content, which allowed for changes in the MA of Met to be reflective of the inclusion level of ChM and peas (Moehn et al., 2005). Vitamin and mineral inclusion levels in the BAS19, ChM63, and PEA63 diets were adjusted based on the contributions of the primary ingredients (i.e., barley and ChM in the ChM63 diet, but only barley in the BAS19 diet) to ensure all nutrients exceeded the Association of American Feed Control Officials (**AAFCO**) recommendations for adult dogs at maintenance (AAFCO, 2023). Due to the differing physicochemical properties of corn starch and its high inclusion level in the BAS diet, processing parameters during extrusion for all three diets were adjusted to achieve comparable specific mechanical energies (HP/hr/ton) (BAS19: 95.3, ChM63: 97.8, PEA63: 97.8) to ensure comparable levels of cook (Gomez and Aguilera, 1984).

**Table 1.** Ingredient composition of the basal (BAS19), chicken (ChM63) and pea (PEA63) treatment diets used to determine the metabolic availability of Met in peas and chicken meal on as-fed (%) basis^1^

	Diet		
Ingredient, %	BAS19	ChM63	PEA63
Green peas, chipped	-	-	37.89
Chicken meal	-	6.58	-
Lamb meal	-	15.00	15.00
Lamb, deboned	-	9.50	9.50
Corn starch	44.88	29.96	-
Barley, pearled	25.82	25.00	25.00
Chicken fat	13.52	6.87	6.96
Beet pulp	3.00	3.00	2.5
Animal digest	1.00	1.00	1.00
Potassium chloride^2^	1.50	0.80	0.11
Salt	1.00	1.00	0.50
Vitamin mix^3^	0.32	0.30	0.26
Mineral mix^3^	0.50	0.30	0.21
Choline chloride	0.40	0.15	0.12
Naturox, dry^4^	0.03	0.03	0.03
Naturox, liquid^4^	0.025	0.025	0.025

^1^Formulation of all three test diets are as formulated and do not reflect any blending of diets. BAS = Corn starch and barley-based reference diet; ChM63 = Chicken meal diet containing chicken meal and lamb as the primary protein sources; PEA63 = Pea diet containing peas and lamb as the primary protein sources.

^2^Minimum purity of 95.6% containing minimum 50% potassium.

^3^Produced by DSM-Firmenich (Kaiseraugst, CH).

^4^Produced by Kemin Industries (Des Moines, IA, USA).

**Table 2.** Analyzed and calculated nutrient and amino acid contents of the basal reference diet with 19% of the Met requirement (BAS), the basal + chicken (ChM40), basal + pea (PEA40), and BAS40 diets with 40% of the Met requirement, and the chicken (ChM63), pea (PEA63), and BAS63 diets with 63% of the Met requirement after blending and supplementation used to determine the metabolic availability of Met in peas and chicken meal on a DM-basis (unless specified)

	Diet														
Item	BAS19		BAS40		ChM40		PEA40		BAS63		ChM63		PEA63		120% of AAFCO rec.^2^
% Met req.^3^	19		40		40		40		63		63		63		-
ME, kcal/kg as-fed^4^ (calculated)	3403		3403		3479		3489		3403		3555		3566		-
Dry Matter, %	88.30		88.30		90.90		90.82		88.30		93.50		93.34		-
Crude Protein, %^5^	23.39 (5.03)		23.39 (5.03)		23.39 (11.29)		23.39 (14.21)		23.39 (5.03)		23.39 (17.54)		23.39		-
Crude Fat, %	15.40		15.40		14.55		14.40		15.40		13.69		13.39		-
Crude Fiber, %	1.40		1.40		1.72		1.78		1.40		2.04		2.16		-
Total dietary fiber, % as-fed	5.93		5.93		7.12		9.37		5.93		8.30		12.80		-
Soluble dietary fiber, % as-fed	1.45		1.45		1.92		2.30		1.45		2.39		3.15		-
Insoluble dietary fiber, % as-fed	4.48		4.48		5.20		7.04		4.48		5.91		9.60		-
NFE, g/100g as-fed^6^ (calculated)	59.77		59.77		56.93		54.97		59.77		54.09		49.94		-
Ash, %	9.3		9.3		8.8		8.2		9.3		8.3		7.0		-
Indispensible AA, %	Diet^7^	Free^8^	Diet	Free	Diet	Free	Diet	Free	Diet	Free	Diet	Free	Diet	Free	
Arg	0.24	0.38	0.24	0.38	0.68	0.19	1.00	0.19	0.24	0.38	1.12		1.76		0.62
His	0.10	0.13	0.10	0.13	0.22	0.06	0.29	0.06	0.10	0.13	0.34		0.49		0.23
Ile	0.18	0.28	0.18	0.28	0.38	0.14	0.51	0.14	0.18	0.28	0.59		0.84		0.46
Leu	0.34	0.48	0.34	0.48	0.76	0.24	0.96	0.24	0.34	0.48	1.18		1.59		0.82
Lys	0.17	0.59	0.17	0.59	0.52	0.29	0.72	0.29	0.17	0.59	0.86		1.27		0.76
Met	0.10		0.10	0.11	0.21		0.21		0.10	0.23	0.32		0.33		-
Phe	0.24	0.77	0.24	0.77	0.46	0.55	0.62	0.39	0.24	0.77	0.69	0.32	1.01		-
Thr	0.17	0.41	0.17	0.41	0.40	0.21	0.50	0.21	0.17	0.41	0.63		0.84		0.58
Trp	0.06	0.13	0.06	0.13	0.11	0.08	0.13	0.06	0.06	0.13	0.16	0.03	0.21		0.19
Val	0.24	0.35	0.24	0.35	0.51	0.18	0.64	0.18	0.24	0.35	0.78		1.04		0.59
Dispensible AA, %	Diet^7^	Free^8^	Diet	Free	Diet	Free	Diet	Free	Diet	Free	Diet	Free	Diet	Free	
Ala	0.22	14.42	0.22	14.35	0.64	9.83	0.72	7.21	0.22	14.29	1.07	5.24	1.21		-
Asp	0.31		0.31		0.80		1.17		0.31		1.29		2.02		-
Cystine^9^	0.11	0.29	0.11	0.29	0.16	0.24	0.20	0.20	0.11	0.29	0.22	0.18	0.30	0.10	-
Tyr	0.18	0.93	0.18	0.93	0.35	0.76	0.46	0.65	0.18	0.93	0.53	0.58	0.74	0.37	-
Glu	0.95		0.95		1.75		2.26		0.95		2.56		3.58		-
Gly	0.22		0.22		0.89		0.88		0.22		1.56		1.55		-
Pro	0.47		0.47		0.92		0.98		0.47		1.36		1.49		-
Ser	0.20		0.20		0.46		0.59		0.20		0.72		0.98		-

^1^ChM40 and PEA40 diets created by blending 50% Basal diet with 50% ChM63 or 50% PEA63 diet to create intermediate level of Met (0.21%). Nutrient analyses presented reflect this blending prior to any additional supplementation with free AA.

^2^Values presented are 120% of the AAFCO minimum recommendations for maintenance of adult dogs on a DM-basis (AAFCO, 2023).

^3^Methionine requirement determined as Met requirement for Labrador Retrievers (0.52 g/100g DM; Mansilla et al., 2020).

^4^ME calculated using modified Atwater calculation.

^5^Presented as: Final Crude Protein content after supplementation with Ala (Crude Protein content of the diet prior to supplementation with Ala).

^6^NFE calculated using NFE, g/100g as-fed = 100—(moisture + Crude Protein prior to supplementation (as-fed) + Crude Fat (as-fed) + Crude Fiber (as-fed) + Ash).

^7^The amount of AA (% DM) that is found in the diet as determined by analytical methods.

^8^The amount of AA (% DM) added as free-AA to ensure the provision of all indispensable AA (except for Met) were provided at at least 120% of the AAFCO recommendations for adult dogs at maintenance (AAFCO, 2023). Additionally, the provision of Tyr, and Phe across all treatments was matched to the PEA63 diet via free-AA supplementation. Cys was supplemented as free-AA to achieve a provision of 0.40 g/100g DM across all diets. For Alanine, this represents the amount of AA (% DM) added to the diet in order to make diets isonitrogenous and match to the crude protein in the PEA63 diet.

^9^Values reported are on a Cystine-basis. However, Cysteine was used as the free-AA as it was easier to work with in solution. Cysteine dosage was determined relative to the mol/kg of Cystine provided in the diet.

All synthetic indispensable AA in the BAS19, ChM63, and PEA63 diets were added as free AA, on an equimolar basis, in a solution of distilled water heated to a maximum of 50 °C to ensure free AA were completely dissolved and then top-dressed on the diet prior to feeding. Phenylalanine (**Phe**) content of all diets was matched to the PEA63 diet which contained over 200% of the AAFCO recommendation for adult dogs at maintenance (AAFCO, 2023) on a DM-basis (1.01% vs 0.45%, respectively; Table 2), and tyrosine (**Tyr**) was provided at 110% of the Phe provision on a DM-basis across all diets. This was to ensure Phe would be shunted to either protein synthesis or oxidation and not to synthesize Tyr (Shiman and Gray, 1998). Cysteine content of all diets was provided at 0.40% DM across all diets to ensure that SAA metabolism would not be limited when Met was provided at 19% of its requirement in the BAS19 diet. This provision was over 60% of the AAFCO (2023) total SAA recommendations (Table 2). All other AA were provided to at least 120% of the AAFCO recommendations for adult dogs on a DM basis to ensure another AA would not limit protein synthesis (AAFCO, 2023). Free alanine (**Ala**) in solution was supplemented when appropriate to make diets isonitrogenous and prepared using the same standards mentioned above. A total of four additional treatments were created to result in three graded levels of Met for the BAS19, ChM63, and PEA63 diets, allowing for a slope-ratio assay approach to be used. For the BAS diet, two additional treatments were created by supplementing Met as DL-Met in solution to achieve 40% and 63% of the requirement (**BAS40**: 0.21% DM and **BAS63**: 0.33% DM; Mansilla et al., 2020). To create the Met levels for the ChM63 and PEA63 diets, they were blended with the BAS19 diet (50% BAS19 and 50% PEA63; **PEA40**, and 50% BAS10 and 50% ChM63; **ChM40**) to achieve Met at 40% of its requirement (0.21% DM; Mansilla et al., 2020). The BAS19 diet was used as the common first point (Moehn et al., 2005).

The study design was a partially replicated 7 × 7 Latin square (*n* = 9), where all dogs were randomly assigned to one of the seven groups of dietary treatments in each experimental period, with no dog receiving the same order of treatments, and ensuring that no treatment was repeated on each calorimetry day per period. During the 14-d diet adaptation period, dogs were fed a commercial extruded wash-in diet (S6 Nutram Sound Balanced Wellness Adult Dog Food, chicken meal and brown rice recipe; Elmira Pet Products, Elmira, ON, Canada) in one meal fed at 0800 hours in amounts known to maintain ideal individual BW based on historical feeding records. Dogs were allowed 15 min to finish their daily meal, and all dogs finished their daily ration during that time. There were seven 7-d experimental periods; the first 2 d were diet adaptation to the treatment diet, followed by the first IAAO trial on d 3 (**D2**), then after 3 d of diet adaptation (6 d total per period), the second IAAO trial occurred on d 7 (**D6**). During the 6-d diet adaptation period, typical IAAO studies require feeding animals the same amount of food in g/kg BW to ensure all dogs are adapted to receiving the same amount of dietary nitrogen (Moehn et al., 2005; Shoveller et al., 2017). However, despite all dogs in this study being of similar ideal BW, their daily energy requirement to maintain ideal BW varied from 1,183 kcal/d to 1,578 kcal/d, which meant that feeding all dogs in this way would result in some dogs undergoing weight gain and some weight loss. As weight gain and weight loss alter metabolism, it was determined that all dogs would continue to be fed in order to maintain ideal BW, as is a principle of IAAO studies, during the 6-d diet adaptation period (Moehn et al., 2005; Shoveller et al., 2017). To control for the variation that this would cause, the daily total ME intake was kept constant for each dog throughout the 6-d adaptation period. Additionally, to ensure that all dogs received the same proportion and balance of AA per total daily amount fed, all free-AA supplementation was dosed as a proportion of the diet. On IAAO breath collection days, food intake was restricted to 13 g/kg BW, which varied between 100% and 76% of the historical feeding allowance known to maintain ideal BW. After completion of each IAAO day, dogs that had not received their total daily feed allowance received the remainder to maintain ideal BW. This feeding protocol ensured that dogs consumed all the test diets during IAAO and received equivalent isotope delivery. After each 7-d experimental period, dogs were fed the commercial wash-in diet for 7 d as described above to ensure that extended feeding of Met-deficient diets would not impact Met and SAA metabolism before dogs began the next experimental period. This feeding protocol was repeated seven times until all dogs received all treatments.

Blood samples (5 mL) were collected from each dog within 30 min after their last meal at the end of each IAAO day from the cephalic vein in a 10 mL sodium heparin tube (Becton Dickinson Canada Inc., Mississauga, ON) and placed on ice. Once all samples were collected, 1 mL of whole blood was separated and stored at −80 °C for analysis of whole blood AA. The other 4 mL of blood was centrifuged at 4 °C at 15,000 × g for 15 min. Plasma was separated, and then all samples were stored in a −80 °C freezer until analysis of fed-state plasma and whole blood AA concentrations.

### Indicator amino acid oxidation study

Each IAAO study day was conducted according to Crosbie et al. (2024), where dogs were first weighed to determine feed, AA solution, and isotope dosing, after which dogs were moved to individual oxidation chambers to which they had already been acclimated. After 30 min of gas equilibration, triplicate resting volume of expired CO_2_ and O_2_ (**VCO**_**2**_; **VO**_**2**_, respectively) measurements were taken. Fasted state-VCO_2_ and VO_2_ measurements were taken due to the increased variability associated with fed state-VCO_2_ in free-living animals. Dogs were then fed (Time 0) their corresponding feed allowance divided into 13 equal small meals, where the first three meals were fed every 10 min to induce a fed state, and the other ten meals were fed every 25 min. The total amount of feed fed during the IAAO study was based on BW measured the same morning after 22 h of fasting (13 g/kg BW). Background ^13^C enrichment was determined by collecting CO_2_ samples over three consecutive 25-min periods. The sixth meal (95 min after first feeding) contained a priming dose (9.4 mg/kg BW) of L-[1-^13^C]-Phe (99%; Cambridge Isotope Laboratories, Inc., Tewksbury, MA, United States of America) and a priming dose (0.176 mg/kg BW) of NaH^13^CO_3_ (99%; Cambridge Isotope Laboratories, Inc.) to prime the bicarbonate pool and reduce the time to reach isotopic steady state (Shoveller et al., 2017; Pezzali et al., 2023a). To maintain the supply of L-[1-^13^C]-Phe, the following seven meals contained constant doses (2.4 mg/kg BW) of L-[1-^13^C]-Phe for all dogs. Expired CO_2_ was collected over the last eight 25-min periods. Overall, during each IAAO study, each dog spent ~6.3 h inside the oxidation chamber. Additional details regarding the timeline for each IAAO study can be found in Mansilla et al. (2018).

### Sample collection and analysis

Expired CO_2_ in breath and calorimetry data was collected and analyzed according to Crosbie et al. (2024) using an open circuit calorimetry system that pulled fresh air into the oxidation chambers by a rotary vane vacuum pump through a series of DRIERITE-filled columns (calcium sulfate impregnated with colbalt chloride as an indicator; W. A. Hammond DRIERITE Co. Ltd) to the CO_2_ analyzer (Qubit Model S155, Quibit Systems Inc., Kingston, ON, Canada) and the gas switcher. From the gas switcher, the expired breath was pushed through midget bubblers, filled with 8 mL of 1 mol/L sodium hydroxide (**NaOH**) solution. The NaOH solution was used to trap CO_2_ released by the dogs while in the oxidation chambers for the subsequent ^13^CO_2_ enrichment analysis (Shoveller et al., 2017). Breath samples were then stored in an air-tight serum tube and kept at room temperature until further analysis. Calorimetry data were collected automatically using Qubit calorimetry software (C950-Multi Channel Gas Exchange System and Software for Animal Respirometry; Qubit Systems Inc., Kingston, ON, Canada). Analysis of ^13^C enrichment in breath samples was conducted at the Environmental Isotope Laboratory, University of Waterloo (200 University Ave W, Waterloo, ON). Breath samples were analyzed with a Gasbench II interfaced with a Delta V Plus mass spectrometer (Thermo Fisher Scientific, Bremen, Germany).

Free AA concentrations in plasma and whole blood were analyzed using ultraperformance liquid chromatography (**UPLC**) to better understand the response to oxidation and any impacts on macronutrient partitioning from the test diets. In brief, 100 μL of plasma or whole blood was deproteinized using 100 μL of 10% sulfosalicylic acid, vortexed, and then centrifuged at 14,000 × *g* for 5 min. The supernatant was derivatized by an ACCQTag Ultra derivatization kit (Waters Corporation, Milford, MA, United States of America). Derivatized AA were separated in a column (2.1 mm × 100 mm, particle size = 1.7 μm) maintained at 55 °C with the use of UPLC (Waters Corporation, Milford, MA, United States of America) with UV detection (260 nm). AA peak areas were compared with known standards and analyzed with Waters Empower 2 Software (Waters Corporation, Milford, MA, United States of America). Total plasma Cys, homocysteine (**Hcys**), and glutathione (**GSH**) were analyzed using UPLC using an adapted method from Vester and Rasmussen (1991) and Pfeiffer et al. (1999) according to Crosbie et al. (2024).

Nutrient contents of the BAS19, ChM63, and PEA63 diets were analyzed for DM, crude protein, crude fat, crude fiber, total dietary fiber, soluble dietary fiber, insoluble dietary fiber, ash, and all AA at the commercial laboratory Eurofins Microbiology Laboratories (Madison, Wisconsin, United States of America). Amino acids in the BAS19, ChM63, and PEA63 diets, except for Trp, Met, cystine, and taurine (**Tau**), were analyzed using the acid hydrolysis procedure (AOAC, 2005; 982.30). Methionine and cystine were quantified using the oxidative hydrolysis procedure (AOAC, 2005; method 994.12), Trp was analyzed using the alkaline hydrolysis procedure (AOAC, 2005; method 988.15), and Tau was analyzed using method 999.12 (AOAC, 2005). Nutrient contents of the PEA40 and ChM40 diets were determined by calculating the amount of each nutrient provided by each of the BAS19 and PEA63 and BAS19 and ChM63 diets, respectively, after blending them in the proportions previously described.

### Calculations

The fraction of ^13^CO_2_ released per kg of BW per h (F^13^CO_2_/kg BW/h) was calculated using the following equation:


$$\begin{array}{l} F^{13}CO_{2}\left(mmol/kg/h\right)=\frac{\left(VCO2\right)\left(APE\right)\left(44.6\right)\left(60\right)}{\left[\left(BW\right)\left(1.0\right)\left(100\right)\right]} \end{array}$$

Where VCO_2_ is the average production of CO_2_ from all breath collection days within each dog at rest in mL/min; APE (atom percent excess) is the average ^13^CO_2_ enrichment in expired breath at isotopic steady state in percent; and BW is the weight of the dog in kg. The constants 44.6 (mmol/mL) and 60 (min/h) convert the VCO_2_ to micromoles per hour; the factor 100 changes APE to a fraction; and the 1.0 is the retention factor of CO_2_ in the body due to bicarbonate fixation as reported previously (Shoveller et al., 2017).

The MA of Met was calculated following the equation by Littell et al. (1997):


$$\begin{array}{l} Metabolic\;availability\;=\frac{bT}{bR} \end{array}$$

where bT and bR were the slopes from the IAAO response (i.e., F^13^CO_2_) following graded intake of Met from the pea and ChM diets, and crystalline AA reference diet.

Resting and fed energy expenditure (**REE, FEE**) were calculated by the C950-Multi Channel Gas Exchange system (Qubit Systems Inc., Kingston, ON, Canada), based on VO_2_ and VCO_2_ using the modified Weir equation (Weir, 1949):


$$\begin{array}{l} Energy\;expenditure\;(kcal/d)\;=\;3.94{\left(VO_{2}\right)}_{}+\;1.11\left(VCO_{2}\right) \end{array}$$

In which VO_2_ and VCO_2_ are the volume of oxygen consumed by the dog and the volume of carbon dioxide produced (L/d), and energy expenditure (kcal/d) was expressed in relation to metabolic BW (BW^0.75^; **mBW**) for all dogs. Resting and fed respiratory quotient (**RQ**) was calculated directly by the Qubit calorimetry software as CO_2_ production/O_2_ production. Fed fat and carbohydrate oxidation (g/min/kg BW) were calculated using the equations established by Frayn (1983):


$$\begin{array}{l} Carbohydrate\;oxidation\;\left(g/min/kg\;BW\right)=\frac{4.59\left(VCO2\right)\;-3.22\left(VO2\right)}{BW} \end{array}$$

$$\begin{array}{l} Fat\;oxidation\;\left(g/min/kg\;BW\right)\;=\frac{1.70\left(VCO2\right)\;-1.70\left(VO2\right)}{BW} \end{array}$$

In which VCO_2_ and VO_2_ are the volume of carbon dioxide produced and volume of oxygen consumed by the dog (L/min) and BW is the weight of the dog in kg.

### Statistical analysis

Statistical analyses were conducted using SAS (Version 9.3, SAS Institute Inc., Cary, NC, United States of America). Plasma and whole blood AA, BW, metabolic BW, REE, FEE, fasted and fed RQ, fat oxidation, and carbohydrate oxidation were analyzed using the PROC GLIMMIX procedure. Diet, adaptation period (D2 or D6), and their interaction were included as fixed effects, while dog and period were treated as random effects. Data are expressed as least squares means ± SEM, and means were separated using the Tukey-Kramer post-hoc test. Methionine intake was expressed as the % of Met from the estimated requirement determined by Mansilla et al. (2020) for Labrador Retrievers (0.52% DM), which were of similar size to those used in this study. Regression within the analysis of variance was determined using PROC GLIMMIX to construct the regression equation for each of the dietary treatments, BAS reference, ChM, and PEA test diets, to obtain the slopes of the three lines. The effect of Met inclusion, day IAAO was conducted (D2 or D6), the addition of Met via ChM, pea, or crystalline AA inclusion, and their interactions on the variation of F^13^CO_2_ were tested using PROC GLIMMIX, with dog and period as random effects. This procedure also tested whether F^13^CO_2_ slopes were significantly different from zero. F^13^CO_2_ results were expressed as a regression equation. Results were considered statistically significant at *P* ≤ 0.05, and *P* > 0.05 was considered not significant.

## Results

All dogs remained healthy and maintained their ideal BW throughout the study. Six dogs completed all 7 experimental periods and consumed all meals on both IAAO collection days. Three dogs did not complete certain treatments, which are summarized below, as they did not consume all 13 meals during the IAAO breath collection day and, therefore, they did not reach isotopic steady state, which is required for IAAO studies (Moehn et al., 2005; Shoveller et al., 2017). Of the three dogs, one dog completed all treatments on both IAAO days except for BAS40 and BAS63; another dog completed all treatments on both IAAO days except for BAS19, BAS40, and D6 of BAS63; and the last dog completed all treatments on both IAAO days except for BAS19, BAS40, and BAS63. This resulted in a total of nine observations for the PEA63, ChM63, ChM40, and PEA40 diets, seven observations for the BAS19 diet, and six observations for the BAS40 and BAS63 diets.

### MA of Met in peas and chicken meal

**Figure 1.**
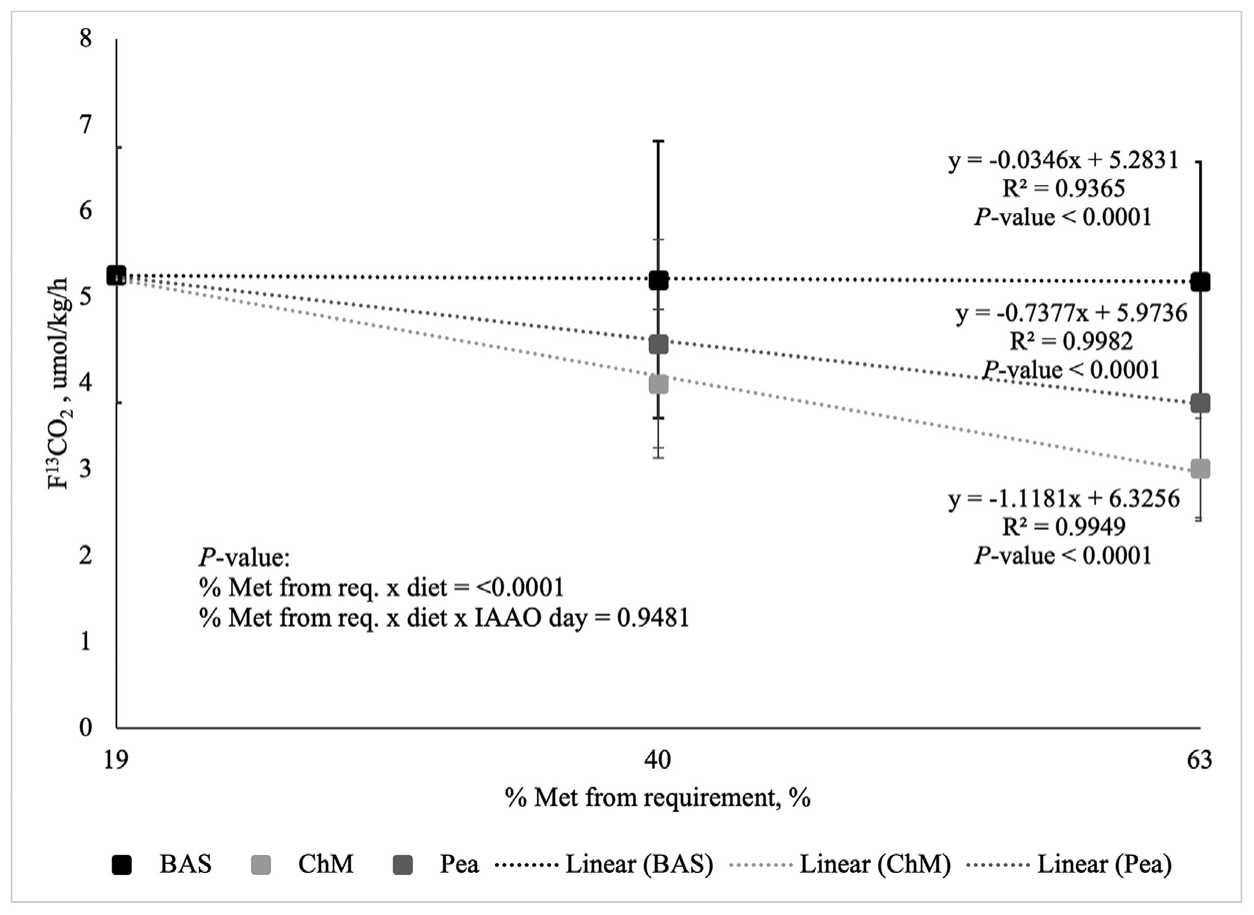
Linearity of the rate of L-[1-^13^C]-Phe oxidation (F^13^CO_2_) in response to graded intake of protein-bound Met from crystalline amino acids (BAS), chicken meal (ChM), and peas in adult dogs from indicator amino acid oxidation conducted after a 2-d diet adaptation (D2). Methionine from BAS was provided at 19 (*n* = 7), 40 (*n* = 6), and 63% (*n* = 7) of the requirement for Labrador Retrievers (0.52% DM), respectively. Methionine from both ChM (*n* = 9) and peas (*n* = 9) was provided at both 40% and 63% of the requirement for Labrador Retrievers (0.52% DM).

**Figure 2.**
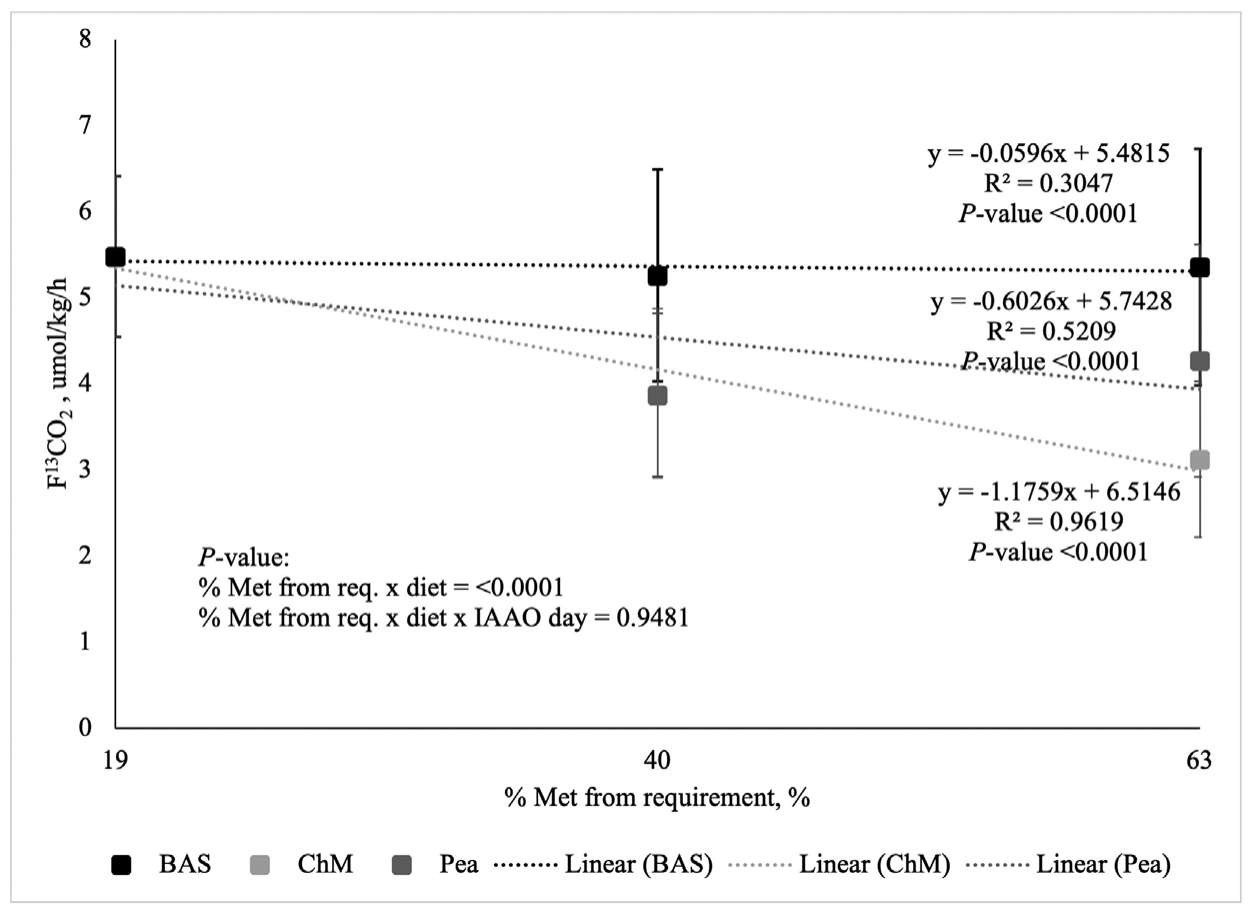
Linearity of the rate of L-[1-^13^C]-Phe oxidation (F^13^CO_2_) in response to graded intake of protein-bound Met from crystalline amino acids (BAS), chicken meal (ChM), and peas in adult dogs from indicator amino acid oxidation conducted after a 6-d diet adaptation (D6). Methionine from BAS was provided at 19 (*n* = 7), 40 (*n* = 6), and 63% (*n* = 7) of the requirement for Labrador Retrievers (0.52% DM), respectively. Methionine from both ChM (*n* = 9) and peas (*n* = 9) was provided at both 40% and 63% of the requirement for Labrador Retrievers (0.52% DM).

The increasing concentration of Met, the different diet types, and the interaction between the two were sources of variation for F^13^CO_2_ (*P* < 0.05; Figures 1 and 2). The interaction between the concentration of Met, diet type, and day of IAAO was conducted for F^13^CO_2_ was determined using the test for regression within the analysis of variance and was not different (*P* = 0.9481). As Met intake from the crystalline AA reference diets (i.e., BAS19, BAS40, and BAS63) increased from 19% to 63% of the requirement for Met (0.52% DM; Mansilla et al., 2020), the rate of ^13^C-Phe oxidation (F^13^CO_2_) decreased linearly. A negative slope of the best-fit line of -0.0346 ± 0.01 (*P* < 0.0001) on D2 (Figure 1) and −0.0596 ± 0.01 (*P* < 0.0001) on D6 (Figure 2) was determined using linear regression for the BAS crystalline AA reference protein. As Met from the common first point and ChM increased from 19% to 63% of the requirement for Met (0.52% DM; Mansilla et al., 2020), the rate of ^13^C-Phe oxidation (F^13^CO_2_) decreased linearly. A negative slope of the best-fit line of −1.1181 ± 0.01 (*P* < 0.0001) on D2 (Figure 1) and −1.1759 ± 0.01 (*P* < 0.0001) on D6 (Figure 2) was determined using linear regression for ChM. As Met from the common first point and peas increased from 19% to 63% of the requirement of Met (0.52% DM; Mansilla et al., 2020), the rate of ^13^C-Phe oxidation (F^13^CO_2_) decreased linearly. A negative slope of the best-fit line of −0.7377 ± 0.01 (*P* < 0.0001) for D2 (Figure 1) and −0.6026 ± 0.01 (*P* < 0.0001) for D6 (Figure 2) was determined using linear regression for peas. However, as the slope of the rate of ^13^C-Phe oxidation (F^13^CO_2_) was greater (i.e., more negative) for both peas and ChM than the semi-synthetic BAS crystalline AA reference protein, the MA of Met in both peas and ChM could not be determined. Despite this, the MA of Met in peas is 66% and 51% of ChM after D2 and D6 of adaptation, respectively, by dividing the slope of oxidation from peas by the slope of oxidation from ChM.

### Body weight and calorimetry data

Body weight and calorimetry data are presented in Table 3. There was an effect of IAAO day on BW and mBW with dogs on IAAO D2 having on average a 0.54% and 0.37% greater BW and mBW, respectively, than those on D6 (*P* < 0.05). There was no difference in REE across all diets (*P *= 0.592), and there was a diet effect for FEE across both IAAO D2 and D6. However, when dietary treatment means were separated by IAAO day, no differences were detected on D2 or D6 (*P* < 0.05). There was a diet effect for fasted RQ where on D2 dogs fed the BAS19, BAS40, BAS63, and ChM40 diets had a greater fasted RQ than dogs fed both the ChM63 and PEA63 diets (*P* < 0.0001), while dogs fed the PEA40 diet only had a greater fasted RQ than those fed the PEA63 diet (*P* < 0.0001). On D6, dogs fed the BAS19, BAS40, and BAS63 diets had a greater fasted RQ than those fed the ChM40, PEA40, ChM63, and PEA63 diets (*P* < 0.0001). There was an effect of diet on fed RQ where on D2, dogs fed the BAS19, BAS40, BAS63, and ChM40 diets had a greater fed RQ than those fed the PEA63 diet, while dogs fed the BAS63 diet had a greater fed RQ than those fed both the ChM63 and PEA63 diets and (*P* < 0.0001). On D6, dogs fed the BAS19 and BAS40 diets had a greater fed RQ than those fed the ChM63 and PEA63 diets, while those fed the BAS63 diet only had a greater fed RQ than those fed the PEA63 diet (*P* < 0.0001). For fat oxidation, there was also an effect of diet on D2, where dogs fed the PEA63 diet had greater fat oxidation than those consuming the ChM40, BAS63, and BAS19 diets (*P* < 0.0001). On D6, dogs fed the PEA63, ChM63, BAS63, PEA40, ChM40, and BAS19 diets had greater fat oxidation than those fed the BAS40 diet, while those fed the PEA63 diet only had greater fat oxidation than those consuming the BAS40 and BAS19 diets (*P* < 0.0001). Additionally, there was an effect of diet on carbohydrate oxidation, where on D2, dogs fed the BAS19, BAS40, BAS63, and ChM40 diets had greater carbohydrate oxidation than those fed the PEA63 diet, while those fed the BAS63 diet had greater carbohydrate oxidation than those fed both the ChM63 and PEA63 diets (*P* < 0.0001). On D6, dogs fed the BAS19, BAS40, and BAS63 diets had greater carbohydrate oxidation than those fed the PEA63 diet, while those fed the BAS19 and BAS40 diets showed greater carbohydrate oxidation than those fed both the ChM63 and PEA63 diets (*P* < 0.0001).

**Table 3.** Bodyweight (BW, kg), metabolic bodyweight (mBW, kg), and indirect calorimetry data for all adult large breed dogs used after indicator amino acid oxidation

	Diet^1^								*P*-value		
Item	BAS19	BAS40	ChM40	PEA40	BAS63	ChM63	PEA63	SEM^2^	Diet	Day	Diet × Day
*n*	7	6	9	9	7	9	9				
% Met req.	19	40	40	40	63	63	63				
BW, kg											
D2^A^	26.8	26.5	26.8	26.8	26.9	26.5	26.7	0.87	0.056	0.041	0.958
D6^B^	26.6	26.4	26.7	26.7	26.6	26.4	26.6	0.87			
mBW, kg^3^											
D2^A^	11.8	11.7	11.8	11.8	11.8	11.7	11.7	0.29	0.058	0.039	0.956
D6^B^	11.7	11.7	11.7	11.8	11.7	11.7	11.7	0.29			
Resting EE, kcal/kg^0.75^											
D2	101.4	97.0	97.4	92.9	103.0	97.6	100.6	4.63	0.592	0.094	0.201
D6	100.0	103.1	101.2	100.2	99.7	103.8	98.5	4.64			
Fed EE, kcal/kg^0.75^											
D2	110.6^a,b^	108.1^a,b^	107.3^a,b^	108.0^a,b^	110.6^a,b^	104.7^b^	106.2^a,b^	3.36	0.0004	0.359	0.609
D6	111.1^a^	109.7^a,b^	110.2^a,b^	107.8^a,b^	109.0^a,b^	106.3^a,b^	105.6^a,b^	3.36			
Fasted RQ											
D2	0.891^a^	0.865^a^	0.853^a^	0.835^a,b^	0.890^a^	0.805^b^	0.779^c^	0.014	<0.0001	0.356	0.131
D6	0.905^a^	0.893^a^	0.822^b^	0.815^b^	0.884^a^	0.788^b^	0.773^b^	0.015			
Fed RQ											
D2	0.867^a,b^	0.850^a,b^	0.852^a,b^	0.836^a,b,c^	0.873^a^	0.812^b,c^	0.790^c^	0.015	<0.0001	0.780	0.445
D6	0.872^a^	0.878^a^	0.831^a,b,c^	0.834^a,b,c^	0.852^a,b^	0.813^b,c^	0.786^c^	0.015			
Fat oxidation, g/min/kg BW											
D2	0.094^b^	0.103^a,b^	0.098^b^	0.112^a,b^	0.089^b^	0.124^a,b^	0.139^a^	0.011	<0.0001	0.724	0.491
D6	0.089^b^	0.083^c^	0.115^a,b^	0.111^a,b^	0.101^a,b^	0.127^a,b^	0.144^a^	0.011			
Carbohydrate oxidation, g/min/kg BW											
D2	0.308^a,b^	0.279^a,b^	0.264^a,b^	0.251^a,b,c^	0.318^a^	0.204^b,c^	0.165^c^	0.027	<0.0001	0.591	0.436
D6	0.347^a^	0.337^a^	0.249^a,b,c^	0.248^a,b,c^	0.289^a,b^	0.207^b,c^	0.156^c^	0.027			

^1^BAS19 = Corn starch and barley-based reference diet containing crystalline AA providing Met at 19% of the requirement; BAS40 = Corn starch and barley-based reference diet containing crystalline AA providing Met at 40% of the requirement; ChM40 = blend of the BAS and ChM63 diets providing Met at 40% of the requirement; PEA40 = blend of the BAS and PEA63 diets providing Met at 40% of the requirement; BAS63 = Corn starch and barley-based reference diet containing crystalline AA providing Met at 63% of the requirement; ChM63 = Chicken diet containing chicken and lamb as the primary protein sources providing Met at 63% of the requirement; PEA63 = Pea diet containing peas and lamb as the primary protein sources providing Met at 63% of the requirement.

^2^Greatest value for SEM.

^3^mBW = BW^0.75.

^A,B^Within each amino acid, rows not showing a common superscript differ (*P* < 0.05) due to day.

^a,b,c,d,e^Values with different letters within the same row differ (*P* < 0.05) due to diet.

### Plasma and whole blood AA

The results for plasma and whole blood AA after each IAAO sampling day are presented in Tables 4 and 5. There was an effect of diet for most of the analyzed AA that generally followed one of two patterns; 1) AA provided in the greatest amounts as free-AA typically resulted in greater concentrations of those AA in plasma and whole blood (i.e., in the BAS19, BAS40, and BAS63 diets) and 2) AA that were provided in greater amounts than in other dietary treatments typically resulted in greater concentrations of the AA in plasma and whole blood (i.e., the PEA63 diet followed by the PEA40, ChM63, and ChM40 diets). For the purposes of brevity, only the AA involved with sulfur AA are described.

**Table 4.** Fed state plasma amino acid concentrations in adult large mixed-breed dogs (nmol/mL)

	Diet^1^								*P*-value		
Item	BAS19	BAS40	ChM40	PEA40	BAS63	ChM63	PEA63	SEM^2^	Diet	Day	Diet × Day
n	7	6	9	9	7	9	9				
% Met req.	19	40	40	40	63	63	63				
Cys											
D2^A^	121.7^c^	127.5^c^	130.9^c^	134.8^b,c^	131.1^c^	142.5^a,b^	153.1^a^	5.00	<0.0001	0.0002	0.532
D6^B^	116.6^c^	118.0^b^	128.9^b^	134.1^a^	121.7^b^	132.3^b^	146.4^a^	5.00			
Hcys											
D2	5.2^c^	6.0^c^	5.6^c^	6.6^b,c^	7.2^b^	7.3^b^	8.8^a^	0.61	<0.0001	0.154	<0.0001
D6	6.2^b^	6.6^b^	6.4^b^	7.3^a,b^	7.9^a^	6.0^b^	7.8^a^	0.61			
Gsh											
D2	12.0^a^	12.5^a^	10.2^a,b^	8.6^b^	12.3^a^	9.8^a,b^	9.2^b^	0.89	<0.0001	0.091	0.100
D6	12.6^a,b^	13.4^a^	11.3^a,b^	11.1^a,b^	12.8^a,b^	8.7^c^	8.9^c^	0.96			
Met											
D2	21.4^e^	47.2^b^	28.8^d,e^	26.8^e^	76.5^a^	39.1^b,c^	35.3^c,d^	2.20	<0.0001	0.292	0.743
D6	22.5^e^	47.5^b^	31.0^d,e^	28.6^e^	73.6^a^	42.0^b,c^	36.7^c,d^	2.20			
Indispensible											
Arg											
D2	82.2^d^	75.9^d^	92.3^d^	126.6^b,c^	84.5^d^	106.3^c,d^	182.0^a^	10.10	<0.0001	0.657	0.289
D6	85.5^d^	58.9^e^	100.8^c,d^	142.1^b^	72.7^d^	112.1^b,c^	189.5^a^	10.13			
His											
D2	82.0^d^	82.3^c,d^	86.5^a,b^	93.4^a,b^	83.2^b,c^	91.4^a,b^	95.8^a^	3.23	<0.0001	0.852	0.943
D6	83.0^c^	81.4^c^	88.6^a,b^	94.3^a^	80.4^b,c^	92.8^a,b^	95.5^a^	3.23			
Ile											
D2	83.5^a^	71.8^a,b^	65.9^a,b^	57.9^b^	77.7^a,b^	55.4^b^	77.9^a,b^	6.06	<0.0001	0.585	0.199
D6	83.4^a^	85.2^a^	63.2^a,b^	67.7^a,b^	66.4^a,b^	61.8^a,b^	72.6^a,b^	6.06			
Leu											
D2	134.2^a^	115.2^a,b^	107.8^b^	107.1^b^	122.1^a,b^	104.8^b^	121.8^a,b^	5.73	<0.0001	0.865	0.153
D6	134.6^a^	128.1^a,b^	107.3^b^	111.8^b^	108.1^b^	109.4^b^	116.4^a,b^	5.74			
Lys											
D2^B^	148.9^a,b^	133.6^b,c^	122.1^c^	149.2^a,b^	142.6^a,b^	116.8^c^	162.5^a^	8.26	<0.0001	0.004	0.584
D6^A^	160.5^a^	150.8^a,b^	129.7^b,c^	158.8^a^	141.4^a,b^	122.0^c^	164.3^a^	8.27			
Phe											
D2	100.3^a^	92.3^a,b^	89.6^a,b^	87.5^c^	98.9^a^	82.3^c^	81.8^c^	5.10	<0.0001	0.074	0.560
D6	102.5^a,b^	105.0^a^	90.4^a,b,c^	90.0^a,b,c^	98.0^a,b,c^	87.3^b,c^	81.9^c^	5.11			
Thr											
D2^B^	165.3^a^	142.4^b^	112.5^c^	125.8^b,c^	155.3^a,b^	96.0^c^	127.3^b,c^	10.21	<0.0001	<0.0001	0.003
D6^A^	221.6^a^	182.4^b^	131.2^c^	146.5^b^	167.7^b,c^	102.4^c^	130.7^c^	10.23			
Trp											
D2	123.4^a,b^	115.1^b^	117.4^b^	123.6^a,b^	119.0^b^	129.8^a,b^	138.1^a^	6.03	<0.0001	0.605	0.930
D6	122.4^b,c^	120.1^b,c^	116.3^c^	121.2^b,c^	120.0^b,c^	132.3^a,b^	140.5^a^	6.04			
Val											
D2	212.5^a^	196.6^a,b^	187.4^b^	183.1^b^	210.4^a^	175.0^b^	189.4^a,b^	7.18	<0.0001	0.210	0.120
D6	221.5^a^	212.7^a,b^	186.6^b,c^	188.1^b,c^	192.6^b,c^	183.0^c^	190.3^b,c^	7.18			
Dispensible											
Ala											
D2	1798.9^a^	1896.3^a^	1044.3^b^	724.6^c^	2086.8^a^	638.0^c^	349.1^d^	79.13	<0.0001	0.662	0.419
D6	1942.0^a^	1905.1^a^	1092.6^b^	776.6^c,d^	1924.8^a^	652.0^d^	343.5^e^	79.23			
Asn											
D2^B^	56.8^c^	54.9^c^	54.1^c^	74.8^b^	57.5^c^	58.2^c^	92.7^a^	2.69	<0.0001	0.005	0.615
D6^A^	60.4^c^	56.5^c^	57.0^c^	80.3^b^	56.2^c^	61.0^c^	98.3^a^	2.70			
Asp											
D2	9.7^a,b^	11.0^a^	9.4^a,b^	8.6^b^	11.0^a^	9.0^b^	8.2^b^	0.46	<0.0001	0.212	0.569
D6	10.0^a^	10.4^a^	9.0^a,b^	8.9^a,b^	10.7^a^	8.2^b^	8.1^b^	0.46			
Cystine											
D2^A^	13.8^c^	14.7^c^	14.6^c^	15.6^b,c^	16.5^a,b,c^	17.4^a,b^	19.4^a^	2.13	<0.0001	0.0006	0.406
D6^B^	12.7^c^	12.1^c^	13.7^c^	14.9^b,c^	13.2^c^	17.2^a,b^	18.4^a^	2.13			
Tyr											
D2	186.5^a^	133.8^b^	114.8^b,c^	102.7^c,d^	164.6^a^	96.7^d,e^	81.8^e^	6.91	<0.0001	0.325	0.006
D6	175.9^a^	159.5^a^	113.6^b^	103.6^b^	160.6^a^	100.5^b^	81.8^c^	6.61			
Glu^3^											
D2^B^	52.0	56.2	52.9	52.1	54.1	51.3	55.4	2.84	0.256	<0.0001	0.318
D6^A^	56.7	55.8	56.1	58.2	56.3	54.8	57.1	2.85			
Gly											
D2^B^	153.4^c^	145.5^c^	222.6^b^	206.1^b^	157.3^c^	270.0^a^	257.3^a^	8.03	<0.0001	0.005	0.338
D6^A^	165.0^d^	154.6^d^	217.9^c^	224.6^c^	156.4^d^	286.5^a^	269.0^a,b^	8.03			
Pro											
D2^B^	169.8^c,d^	161.5^d^	209.1^a,b^	193.2^b,c^	179.7^c,d^	231.9^a^	211.6^a,b^	8.01	<0.0001	0.011	0.553
D6^A^	178.6^c^	169.4^c^	215.1^b^	205.3^b^	172.5^c^	246.8^a^	220.8^b^	8.03			
Ser											
D2	154.5^a^	131.1^a,b^	137.3^a,b^	133.4^a,b^	147.9^a,b^	130.0^a,b^	123.7^b^	8.38	<0.0001	0.171	0.282
D6	165.8^a^	138.1^a,b^	142.4^a,b^	142.4^a,b^	131.0^b^	135.6^b^	131.3^b^	8.39			
Tau											
D2^A^	64.1^b^	68.5^b^	78.9^b^	81.1^b^	77.9^b^	101.8^a^	108.2^a^	5.36	<0.0001	<0.0001	0.419
D6^B^	56.3^c^	58.1^c^	61.8^c^	76.9^b^	59.5^c^	91.9^a^	96.8^a^	5.01			

^1^BAS19 = Corn starch and barley-based reference diet containing crystalline AA providing Met at 19% of the requirement; BAS40 = Corn starch and barley-based reference diet containing crystalline AA providing Met at 40% of the requirement; ChM40 = blend of the BAS and ChM63 diets providing Met at 40% of the requirement; PEA40 = blend of the BAS and PEA63 diets providing Met at 40% of the requirement; BAS63 = Corn starch and barley-based reference diet containing crystalline AA providing Met at 63% of the requirement; ChM63 = Chicken diet containing chicken and lamb as the primary protein sources providing Met at 63% of the requirement; PEA63 = Pea diet containing peas and lamb as the primary protein sources providing Met at 63% of the requirement.

^2^Greatest value for SEM.

^3^Reported Glu concentrations are the sum of both Glu and citrulline as these co-elute using the UPLC method.

^A,B^Within each amino acid, rows not showing a common superscript differ (*P* < 0.05) due to day.

^a,b,c,d,e^Values with different letters within the same row differ (*P* < 0.05) due to diet.

**Table 5.** Fed whole blood amino acid concentrations after indicator amino acid oxidation in adult large mixed-breed dogs (nmol/mL)

	Diet^1^								*P*-value		
Item	BAS19	BAS40	ChM40	PEA40	BAS63	ChM63	PEA63	SEM^2^	Diet	Day	Diet × Day
n	7	6	9	9	6	9	9				
% Met req.	19	40	40	40	63	63	63				
Indispensible											
Arg											
D2	192.8^c^	187.2^c^	189.9^c^	221.6^b^	194.8^c^	212.3^b,c^	264.6^a^	9.19	<0.0001	0.8416	0.124
D6	196.6^b,c^	166.1^c^	207.0^b^	231.0^b^	182.6^c^	212.4^b^	272.7^a^	9.19			
His											
D2	99.2^a,b^	101.0^a,b^	102.4^a,b^	114.9^a^	98.9^a,b^	114.9^a^	117.6^a^	5.32	<0.0001	0.952	0.227
D6	105.1^a,b^	89.3^b^	114.5^a^	113.1^a^	96.9^a,b^	111.3^a,b^	119.6^a^	5.32			
Ile											
D2	94.2^a,b^	81.8^a,b^	79.3^b^	90.3^a,b^	92.2^a,b^	87.7^a,b^	99.9^a^	5.31	0.001	0.762	0.518
D6	97.9	75.8	87.5	93.6	84.4	86.6	96.3	5.31			
Leu											
D2	157.5^a^	133.6^a,b^	128.5^b^	142.3^a,b^	145.7^a,b^	143.4^a,b^	156.7^a^	6.96	<0.0001	0.497	0.401
D6	158.9^a^	125.0^b^	140.3^a,b^	142.3^a,b^	129.4^a,b^	144.0^a,b^	152.8^a,b^	6.96			
Lys											
D2	279.9^a,b^	284.1^a,b^	246.2^b^	279.7^a,b^	279.6^a,b^	262.4^b^	295.4^a^	11.93	<0.0001	0.082	0.032
D6	310.0^a^	257.4^c^	273.9^a,b^	287.3^a,b^	281.4^a,b^	270.6^b,c^	299.9^a,b^	11.84			
Met											
D2	27.4^d^	43.5^b^	31.6^c,d^	31.7^c,d^	67.0^a^	40.1^b^	38.9^b,c^	2.16	<0.0001	0.660	0.153
D6	27.8^d^	39.4^b,c^	35.3^b,c,d^	32.3^c,d^	61.4^a^	41.9^b^	39.2^b,c^	2.16			
Phe											
D2	109.2	98.7	100.1	103.3	108.2	103.1	100.6	5.09	0.092	0.823	0.787
D6	111.7	91.7	106.1	104.7	102.2	101.5	101.6	5.09			
Thr											
D2^B^	187.7^a^	170.8^a,b^	143.6^b^	158.9^a,b^	185.9^a^	137.4^b^	163.2^a,b^	9.03	<0.0001	0.0003	0.011
D6^A^	237.0^a^	178.3^b,c^	168.1^b,c^	173.2^b,c^	184.7^b^	141.9^c^	165.3^b,c^	9.03			
Trp											
D2	58.1	54.7	56.7	59.1	57.6	60.2	61.5	3.85	0.029	0.131	0.719
D6	58.0^a,b^	48.4^b^	58.6^a,b^	57.9^a,b^	53.5^a,b^	57.5^a,b^	60.4^a,b^	3.85			
Val											
D2	215.1^a^	207.1^a^	184.3^b^	197.7^a^	211.2^a^	194.3^a^	206.2^a^	7.71	0.0002	0.556	0.046
D6	222.8^a^	194.1^a,b^	202.1^a,b^	196.4^a,b^	191.1^b^	190.7^b^	205.8^a,b^	7.71			
Dispensible											
Ala											
D2	1455.2^b^	1499.6^b^	850.5^c^	650.6^d^	1825.6^a^	574.0^d^	365.0^e^	62.00	<0.0001	0.955	0.002
D6	1607.0^a^	1525.5^a^	911.4^b^	700.4^c^	1549.1^a^	581.4^c^	355.5^d^	71.34			
Asn											
D2	44.7^b^	44.3^b^	43.4^b^	51.0^a,b^	44.3^b^	48.1^b^	56.3^a^	3.99	<0.0001	0.786	0.374
D6	45.4^b,c^	37.8^d^	47.3^b,c^	50.7^a,b^	42.7^c^	46.7^b,c^	59.1^a^	3.99			
Asp											
D2	59.2	59.0	55.1	60.0	63.3	58.5	62.9	3.29	0.146	0.422	0.385
D6	61.8	53.1	59.6	58.4	58.5	57.2	62.1	3.29			
Cystine											
D2	2.0	2.3	1.9	1.6	1.8	1.7	1.8	0.41	0.529	0.606	0.458
D6	2.0	1.9	2.0	2.2	2.4	1.5	1.6	0.39			
Tyr											
D2	150.5^a^	137.1^a^	121.7^b^	117.7^b^	153.2^a^	110.5^b^	103.5^b^	6.15	<0.0001	0.422	0.296
D6	166.4^a^	132.0^b^	129.9^b^	120.5^b,c^	146.2^a^	109.6^c^	104.2^c^	6.61			
Glu^3^											
D2	155.0^b^	159.3^b^	158.8^b^	175.7^a,b^	165.8^a,b^	169.3^a,b^	189.1^a^	8.93	<0.0001	0.424	0.321
D6	167.3^a,b^	148.0^b^	176.4^a,b^	176.2^a,b^	162.7^a,b^	168.1^a,b^	192.9^a^	8.93			
Gly											
D2	200.4^b,c^	192.7^c^	238.9^b^	238.3^b^	202.0^b,c^	288.6^a^	284.8^a^	9.85	<0.0001	0.191	0.417
D6	211.5^c,d^	177.5^d^	260.7^a,b^	250.4^b,c^	194.0^d^	299.9^a^	294.0^a^	9.85			
Pro											
D2	191.1^c^	183.4^c^	213.0^a,b^	208.1^a,b^	202.2^a,b,c^	237.0^a^	230.7^a,b^	9.62	<0.0001	0.981	0.236
D6	195.5^b,c^	163.9^c^	229.0^b^	217.3^b^	183.6^c^	241.3^a^	235.7^a^	9.62			
Ser											
D2	194.3	175.3	175.5	178.6	195.0	180.4	181.2	8.69	0.006	0.696	0.130
D6	210.0^a^	161.3^b^	189.7^a,b^	185.4^a,b^	174.0^a,b^	185.2^a,b^	185.1^a,b^	8.69			
Tau											
D2^A^	158.3^b^	161.8^a,b^	162.8^b^	175.7^a,b^	169.0^a,b^	184.5^a,b^	190.6^a^	7.49	<0.0001	<0.0001	0.464
D6^B^	135.2^c^	126.5^d^	152.5^a,b^	150.4^a,b^	144.1^a,b^	168.6^a^	173.4^a^	7.49			

^1^BAS19 = Corn starch and barley-based reference diet containing crystalline AA providing Met at 19% of the requirement; BAS40 = Corn starch and barley-based reference diet containing crystalline AA providing Met at 40% of the requirement; ChM40 = blend of the BAS and ChM63 diets providing Met at 40% of the requirement; PEA40 = blend of the BAS and PEA63 diets providing Met at 40% of the requirement; BAS63 = Corn starch and barley-based reference diet containing crystalline AA providing Met at 63% of the requirement; ChM63 = Chicken diet containing chicken and lamb as the primary protein sources providing Met at 63% of the requirement; PEA63 = Pea diet containing peas and lamb as the primary protein sources providing Met at 63% of the requirement.

^2^Greatest value for SEM.

^3^Reported Glu concentrations are the sum of both Glu and citrulline as these co-elute using the UPLC method.

^A,B^Within each amino acid, rows not showing a common superscript differ (*P* < 0.05) due to day.

^a,b,c,d,e^Values with different letters within the same row differ (*P* < 0.05) due to diet.

### Plasma AA

For the plasma AA (Table 4), there was an effect of diet (*P* < 0.0001) and day (*P* < 0.05), but not the interaction between the 2 (*P* = 0.532) for Cys concentrations, with dogs on D2 having greater Cys than those on D6. On D2, the PEA63 diet had greater Cys concentrations than all other dietary treatments except for the ChM63 diet (*P* < 0.0001). Additionally, Cys for the ChM63 diet was greater than the BAS19, BAS40, BAS63, and ChM40 diets (*P* < 0.0001). On D6, the PEA63 and PEA40 diets had the greatest Cys concentrations among all other treatments (*P* < 0.0001). There was an effect of diet (*P* < 0.0001) and the interaction between diet and day (*P* < 0.0001), but no effect of day (*P* = 0.154) for Hcys concentrations. On D2, the PEA63 diet had the greatest Hcys followed by the ChM63, BAS63, PEA40, BAS40, ChM40, and BAS19 diets (*P* < 0.0001). On D6, the PEA63 and BAS63 diets had greater concentrations of Hcys than the ChM63, ChM40, BAS40, and BAS19 diets (*P* < 0.0001) but were not different than the PEA40 diet. For GSH concentrations, there was only an effect of diet (*P* < 0.0001), where on D2, dogs fed the BAS19, BAS40, and BAS63 diets had greater GSH than those fed the PEA40 and PEA63 diets; however, the ChM40 and ChM63 diets were not different. On D6, dogs fed the PEA63 and ChM63 diets had lower concentrations of GSH than all other treatments (*P* < 0.0001).

When means were separated by day, there were no differences in serine (**Ser**) concentrations on D2 despite the effect of diet (*P* < 0.0001); however, on D6, Ser was greater in the BAS19 diet than the BAS63, ChM63, and PEA63 diets (*P* < 0.0001). There was an effect of both diet (*P* < 0.0001) and day (*P* < 0.0001), but not the interaction between the two (*P* = 0.419), on concentrations of plasma Tau, with Tau being greater on D2 than D6. On D2, Tau was greater in the PEA63 and ChM63 diets than all other treatments (*P* < 0.0001), and on D6, Tau was greatest in the PEA63 and ChM63 diets, followed by the PEA40 diet, then lastly by the ChM40, BAS63, BAS40, and BAS19 diets (*P* < 0.0001). There was an effect of both diet (*P* < 0.0001) and day (*P* < 0.05), but not the interaction between the two (*P* = 0.338), on glycine (**Gly**), with concentrations of Gly being greater on D6 than D2. On both D2 and D6, Gly was greatest in the ChM63 and PEA63 diets, followed by the ChM40 and PEA40 diets, then the BAS63, BAS40, and BAS19 diets (*P* < 0.0001).

### Whole blood AA

Methionine concentrations were greater in the BAS63 diet than in all other treatments on D2 and D6 (*P* < 0.0001). Additionally, on D2, Met in the ChM63 and BAS40 diets was greater than in the ChM40, PEA40, and BAS19 diets, and on D6, Met was also greater in the ChM63 diet than in the PEA40 and BAS19 diets (*P* < 0.0001). There was both an effect of diet (*P* < 0.0001) and day (*P* < 0.0001) on whole blood Tau, with Tau being greater on D2 than D6. On D2, Tau was greater in the PEA63 diet than the ChM40 and BAS19 diets (*P* < 0.0001), and on D6, Tau was greater in the PEA63, ChM63, BAS63, PEA40, and ChM40 diets than both the BAS40 and BAS19 diets (*P* < 0.0001). There was an effect of diet (*P* < 0.05) on all whole blood indispensable AA (Table 5) except for Phe, which had no effect of diet (*P* = 0.092), day (*P* = 0.823), or the interaction between the two (*P* = 0.153).

## Discussion

To our knowledge, this study is the first to apply the IAAO technique using an extruded, semi-synthetic reference diet containing supplemental crystalline AA to determine the MA of AA in ingredients or compounded foods fed to dogs. Previously, we attempted to determine the MA of Met in peas using ChM as a reference protein (Crosbie et al., 2024). Chicken meal was selected because it is widely used in commercial pet foods and could be extruded to ensure all nutrients are subjected to the same processing parameters. However, Crosbie et al. (2024) reported that the MA of Met in ChM was less than that of peas and overall, ChM was a poor reference protein due to the high levels of processing it undergoes during rendering and extrusion. Therefore, in this study, we sought to create an extruded animal-protein-free semi-synthetic BAS reference diet that supplied over 78% of crude protein and AA as crystalline AA top-dressed to avoid heat damage. The reference protein must result in lower oxidation of the isotopic tracer (i.e., C^13^-Phe), to enable MA calculation (Littell et al., 1997; Moehn et al., 2005), and in human and pig studies, reference diets are largely based on supplying supplemental AA, which are considered fully digestible and bioavailable (Moehn et al., 2005; Fakiha et al., 2020). The semi-synthetic BAS crystalline AA reference protein did not meet this criterion, as it had a higher rate of oxidation than both ChM and peas, regardless of the day IAAO was conducted preventing true MA determination for either ingredient. Despite this, ChM had a lower level of oxidation than peas, indicating 34% and 49% greater Met MA on IAAO studies conducted on D2 and D6, respectively. The absence of a day effect supports the adequacy of the standard 2-d diet adaptation period for IAAO MA studies. Further, the near-horizontal BAS slope suggests another AA was more limiting than Met. This suggests that providing all other AA at ≥ 120% of AAFCO recommendations may still be insufficient to support protein synthesis in adult large breed mixed hounds. These findings align with Mansilla et al. (2020), who observed breed size differences in the minimum dietary Met requirement, highlighting that AAFCO and NRC AA recommendations may not account for the influence of body size or breed.

As stated previously, IAAO studies used to determine the MA of AA in ingredients require a reference diet containing all indispensable AA at 100% MA and the test AA as the most limiting (Moehn et al., 2005). While mash-type diets are common in other species (Moehn et al., 2005; Tansil et al., 2022; Pezzali et al., 2023b), we formulated an extruded reference diet to reflect the estimated 60% of dogs consuming kibble as their primary diet (Dodd et al., 2020), and top-dressed crystalline AA to ensure similar processing across diets. A primary requirement of all IAAO MA studies is that the test AA (i.e., Met) must be the most limiting AA and, therefore, all other indispensable AA must be provided in excess of requirements (Moehn et al., 2005). The AA recommendations for adult dogs at maintenance outlined by AAFCO (2023) are based on the National Research Council (**NRC**; NRC, 2006) requirements for adult dogs at maintenance and are generally inflated to account for differences in digestibility and bioavailability of AA in ingredients typically used in commercial dog food formulations. Therefore, all indispensable AA in the BAS diets were set to 120% of the AAFCO (2023) recommendations for adult dogs at maintenance. Despite this, the BAS reference diets had a linear negative oxidation slope (-0.0346), which can be considered as showing no response in a biological sense. This could be due to several factors. First, another AA may have been more limiting, driving oxidation across all BAS diets to a similar level, which is expected as all indispensable AAs except Met were provided at identical concentrations among BAS diets (Moehn et al., 2005; Shoveller et al., 2017). Barley was added to enable extrusion, making the BAS reference diets semi-synthetic with over 78% of crude protein from free-AA. Some protein-bound AA from barley, which counted towards the total provision of AA, may have altered oxidation, although Weijzen et al. (2022) reported no differences in mixed muscle protein synthesis rates when protein-bound AA from intact milk protein were fed compared to free AA in humans. Therefore, further research is needed to determine how the provision of free-AA and protein-bound AA may impact the response to oxidation when used together in a reference diet. Second, selecting Ala as the only dispensable AA to make diets isonitrogenous in the BAS reference diets may have been inappropriate. MacLennan et al. (1987) found that perfusion of Gln in the presence of insulin increased protein synthesis by 80% in rats. However, more recently Cooper et al. (2020) used the IAAO technique in humans and found that individual dietary supplementation of all dispensable AA, except for Gln and Pro, but including Ala, improved the rate of F^13^CO_2_ oxidation, and thus protein synthesis, when all indispensable AA were provided at the recommended dietary allowance. Thus, it is likely that the use of Ala to make all BAS reference diets isonitrogenous was appropriate as the IAAO technique is a more accurate measure of amino acid oxidation, and inversely, protein synthesis in vivo (Moehn et al., 2005). Third, the indispensable AA to dispensable AA ratio (IAA:DAA) in the BAS reference diets (0.30) may also have limited protein synthesis, as compared to ChM63 (0.46) and PEA63 (0.76). Cooper et al. (2020) determined that an IAA:DAA of 1 supported synthesis in adult humans when all indispensable AA were provided at the recommended dietary allowance. While there is a dearth of research exploring how the IAA:DAA impacts the rate of protein synthesis in dogs, Gotterbarm et al. (1998) determined that growing pigs fed isonitrogenous diets with an IAA:DAA of 0.40 excreted four times more urinary nitrogen than those fed an IAA:DAA of 1. Given the observed oxidation response from ChM in the current study, it is also possible that the IAA:DAA in the BAS reference diets was not high enough to further support protein synthesis. Therefore, providing Met at up to 63% of the requirement for Labrador Retrievers, determined using the IAAO technique (0.52% DM; Mansilla et al., 2020), and providing all other indispensable AA at 120% of the AAFCO (2023) recommendations did not produce an oxidation response and were insufficient to support protein synthesis in the context of this study. Future research should include reformulation of the BAS reference diets to determine limiting AA and whether increasing the IAA:DAA would also improve protein synthesis in these diets.

Although animal IAAO studies typically use a standard 2-d diet adaptation (Moehn et al., 2005; Shoveller et al., 2017; Mansilla et al., 2020; Tansil et al., 2022), AA ileal digestibility studies typically use an adaptation period of anywhere between 5-8 d to capture effects of ingredient composition, including dietary fiber (Bednar et al., 2000; Crosbie et al., 2020; Cargo-Froom et al., 2023a). Peas contain more total dietary fiber compared to animal-derived ingredients and have been seen to increase losses of ileal endogenous AA in pigs (Leterme et al., 1996). However, no research exists to indicate if high dietary fiber may also decrease AA MA after a greater than 2-d diet adaptation. Therefore, we repeated IAAO after 6-d of adaptation to determine if this would affect the MA of Met in peas. No effect of day was observed, consistent with Szwiega et al. (2023) who found there was no difference in the estimated Thr requirement in adult men using the IAAO technique after 1-d, 3-d, and 7-d of diet adaptation. Together, these findings support the use of a standard 2-d diet adaptation period to determine the MA of AA or to determine AA requirements in animals and humans using the IAAO technique.

Previous work from our laboratory suggested that the MA of Met in ChM was less than that of peas (Crosbie et al. 2024). However, the opposite was found in the present study. The ChM used in these two studies came from different batches. Preparation of ChM for use in pet food requires heating the raw product at a minimum temperature for a minimum amount of time, as outlined by the country the pet food is being produced, to remove water, leading to the elimination of microbial contamination and improvement of shelf-life. However, there is often no maximum for these (NRC, 2006). As the ChM63 diets were formulated and processed nearly identically, differences in the MA of Met in ChM between the two studies were likely due to variability in the temperature and time that each batch of ChM was rendered at, which can impact AA bioavailability (Awonorin et al., 1995; Donadelli et al., 2019; Oba et al., 2019; Geary et al., 2023). Unfortunately, the processing parameters for the rendering of the ChM used in the two studies were unavailable, so they could not be reported. This highlights the variability in MA of AA in ingredients that undergo greater levels of processing and how this may impact dog food formulation using ChM. Understanding the variability in not only AA contents, but also digestibility and bioavailability of ingredients depending on processing is recommended for companies to track and understand to maintain consistency in product formulation and subsequent performance.

Plasma Met concentrations in all diets except for the BAS63 diet were below reported reference ranges for healthy adult dogs, which was expected as Met was provided below the requirement in all diets (Delaney et al., 2003). Despite identical Met provision across the ChM63, PEA63, and BAS63 diets, plasma and whole-blood Met were greater in dogs fed the BAS63 diet, suggesting greater bioavailability and absorption of free AA (Chung and Baker, 1992; NRC, 2006; Mansilla et al., 2020). This effect was also present in whole blood Met. However, greater plasma Met from the BAS63 diet did not result in greater plasma Cys (on D2 and D6) or Hcys (on D2 but not D6) compared to the PEA63 diet. As Cys was provided in identical amounts across all diets, greater plasma GSH from the BAS diets compared to the PEA63 diet on D2 and both the PEA63 and ChM63 diets on D6 suggests that a greater proportion of Cys from the BAS diets was preserved as GSH (Griffith, 1987). Alternatively, there may have been more oxidative products in the PEA63 and ChM63 diets, leading to increased GSH utilization and requiring more Cys to support this increased utilization (Griffith, 1987; Poljsak et al., 2021). Additionally, whole blood Tau was not different between the BAS63, ChM63, and PEA63 diets on either D2 or D6. However, plasma Cys and whole blood Tau were greater on D2 than D6, suggesting time-dependent changes in SAA metabolism with differing sources of Met (Griffith, 1987). Other AA differences in plasma and whole blood were generally reflective of the differences in the provision of those AA in the test diets, with crystalline AA-rich diets generally leading to greater circulating concentrations. Overall, plasma and whole blood AA concentrations across dietary treatments were generally within normal reported ranges found in healthy adult dogs at maintenance and in agreement with previous findings from a similar IAAO MA using the same population of dogs (apart from Ala, Phe, and Tyr, and Met and GSH, which were above and below the normal range, respectively; Delaney et al., 2003; NRC, 2006; Beloshapka et al., 2018; Banton et al., 2021; Crosbie et al., 2024).

Bodyweight and mBW were greater on D2 than D6; however, differences were ≤ 1.1% and likely of little biological relevance. There were no differences in BW and mBW across treatments within each IAAO day, and all dogs maintained their ideal BW throughout the study. Resting and fed EE remained similar across all treatments, which suggests that feeding differing concentrations of dietary Met from both ChM and peas did not affect energy metabolism, as is required for design principles in MA studies on animals at maintenance (Moehn et al., 2005; Shoveller et al., 2017). Dogs fed the PEA63 diets had lower fasted and fed RQ, as well as lower carbohydrate oxidation, on both D2 and D6 compared to dogs fed any of the BAS reference diets. They also had greater fat oxidation on D2. However, on D6, greater fat oxidation was only observed in dogs fed the PEA63 diet compared to those fed the BAS19 and BAS40 diets. These observations may be due to several factors. In terms of diet composition, although the BAS diets contained 14% more crude fat than the PEA63 diet, the BAS diets had nearly 18% greater calculated nitrogen-free extract (**NFE**). Additionally, 18% of the total crude protein contents of the BAS diets were from crystalline AA compared to 23% from intact proteins in the PEA63 diet. The higher fasted and fed RQ observed in dogs fed the BAS diets is likely due to the substantial provision and high bioavailability of AA, as well as higher NFE content, which resulted in a greater proportion of energy coming from carbohydrate and protein (Goldenshluger et al., 2021). Additionally, Yang et al. (1986) found that increased carbohydrate intake led to increased de novo Ala synthesis in humans. Therefore, it is likely that both the increased carbohydrate supplied in the BAS diets and the supplementation of Ala to make all diets isonitrogenous, particularly in the BAS diets (at nearly 15% DM), resulted in a further increase in glycolysis and overall carbohydrate oxidation in these diets (Felig, 1973; Yang et al., 1986). This effect was also observed in a previous study by Crosbie et al. (2024), which found that IAAO diets with higher Ala supplementation resulted in higher fed RQs and carbohydrate oxidation. Studies to determine the MA of limiting AA in ingredients require precise diet design around ensuring diets are isoenergetic, isonitrogenous, and provide precise levels of key dietary AA; however, other macronutrient dietary contents, such as fiber, or macronutrient balance within these parameters, are considered inherent to the ingredients being tested themselves (Moehn et al., 2005). Therefore, it is reasonable that the different macronutrient compositions of the test diets and Ala supplementation shifted macronutrient oxidation.

## Conclusions and Implications

The data presented above suggests that the diet design and execution of the IAAO technique to determine MA of Met in both ChM and peas was successful in creating a linear response in oxidation to graded intakes of protein-bound Met from these ingredients. However, as the slope of oxidation from the BAS reference diet was nearly horizontal (indicating another AA in the BAS reference diet was likely limiting) and greater than that of peas and ChM, the MA of Met in peas could be determined but was less than ChM. As there was no effect of the length of adaptation prior to when IAAO was conducted (D2 versus D6), the standard 2-d diet adaptation was considered appropriate. In order to define an acceptable reference protein formulation, future research should be done to identify the limiting AA in the BAS crystalline AA diet. Overall, further refinement of the reference diet is required to use this method to measure the MA of AA in ingredients fed to dogs.

## Abbreviations

AA: amino acid
AAFCO: Association of American Feed Control Officials
APE: atom percent excess
BW: body weight
ChM: chicken meal
DM: dry matter
FEE: fed energy expenditure
IAAO: indicator amino acid oxidation
IDAA:DAA: indispensable AA to dispensable AA ratio
MA: metabolic availability
mBW: metabolic BW
ME: metabolizable energy
NaOH: sodium hydroxide
NFE: nitrogen-free extract
NRC: National Research Council
REE: resting energy expenditure
RQ: respiratory quotient
SAA: sulfur amino acid
UPLC: ultra performance liquid chromatography
VCO_2_: volume of expired carbon dioxide
VO_2_: volume of expired oxygen
